# Emotional and behavioural problems of left behind children in Lithuania: a comparative analysis of youth self-reports and parent/caregiver reports using ASEBA

**DOI:** 10.1186/s13034-024-00726-y

**Published:** 2024-03-18

**Authors:** Justina Račaitė, Khatia Antia, Volker Winkler, Sigita Lesinskienė, Rita Sketerskienė, Rūta Maceinaitė, Ingrida Tracevskytė, Elena Dambrauskaitė, Genė Šurkienė

**Affiliations:** 1 Department of Public Health, Institute of Health Sciences, Faculty of Medicine, Vilnius University, M.K. Čiurlionio 21, Vilnius, LT-03101 Lithuania; 2 Heidelberg Institute of Global Health, Heidelberg University Hospital, Im Neuenheimer Feld 130/3, 69120 Heidelberg, Germany; 3 School of Health Sciences, The University of Georgia, 77a Kostava Street, Tbilisi, 0175 Georgia; 4 Clinic of Psychiatry, Institute of Clinical Medicine, Faculty of Medicine, Vilnius University, M. K. Čiurlionio g. 21, Vilnius, LT-03101 Lithuania; 5 Institute of Psychology, Faculty of Philosophy, Vilnius University, Universiteto Str. 9, Vilnius, LT-01513 Lithuania

**Keywords:** Left behind, Children, Parents, Emigration, ASEBA, Mental health

## Abstract

**Background:**

Children being left behind (LBC) in their home countries due to parental emigration is a global issue. Research shows that parents’ emigration negatively affects children’s mental health and well-being. Despite a high number of LBC, there is a dearth of data from Eastern European countries. The present study aims to collect and analyse self-reported data on LBC emotional and behavioural problems and compare children’s reports with those of parents/caregivers.

**Methods:**

A cross-sectional study was conducted in 24 Lithuanian schools, involving parents/caregivers and their children aged 12 to 17. We employed self-reported measures, including the Achenbach System of Empirically Based Assessment (ASEBA) tools – Child Behaviour Checklist (CBCL 6/18) and Youth Self Report (YSR 11/18), to evaluate the emotional and behavioural problems of the children. These instruments had been translated, standardised, and validated for the Lithuanian population. Data collection took place between January 2022 and April 2023. In addition to descriptive analysis, multivariate regression was used to adjust for various sociodemographic factors.

**Results:**

A total of 760 parents/caregivers and 728 of their children participated in the study. LBC exhibited higher total problem scores (57.7; 95% CI 52.0-63.4) compared to non-LBC (47.1; 95% CI 44.7-49.4). These differences were consistent across all YSR 11/18 problem scales. However, no significant differences were observed in CBCL 6/18 scores. Furthermore, LBC self-reported a higher total problem score (57.7; 95% CI 52.0-63.4) compared to their parents/caregivers (24.9; 95% CI 18.9-30.9), and this pattern persisted across all scales. Being female, having school-related problems and having LBC status were associated with higher YSR 11/18 scores in the multivariable regression, while female gender, living in rural areas, school-related problems, and having hobbies were associated with higher CBCL 6/18 scores.

**Conclusion:**

This study highlights that LBC report more emotional and behavioural challenges than their non-LBC peers, while parent/caregiver assessments show lower problem scores for LBC. Gender, living environment, school-related issues, and engagement in hobbies have influenced these outcomes. These findings underscore the multifaceted nature of the experiences of LBC and the importance of considering various contextual factors in understanding and addressing their emotional and behavioural well-being.

**Supplementary Information:**

The online version contains supplementary material available at 10.1186/s13034-024-00726-y.

## Introduction

Globally, the estimated count of international migrants reached nearly 281 million in 2020, with labour migration increasing from 164 million in 2017 to 169 million in 2019 [1]. Due to labour migration worldwide, hundreds of millions of children remain in their home countries [2]. In academic literature, the term “children left behind” (LBC) refers to those who stay in their home countries while their parents migrate for work [3, 4]. Parents often face the difficult choice of staying with their children or relocating to improve their children’s well-being. The constraints of parental migration often limit their ability to care for their children, leading LBC to reside with extended family, friends, or even on their own [3].

Though exact number of LBC remains uncertain, prior research has estimated that approximately 36% of Moldovan children and 39% of Georgian children live in households where at least one member has embarked on migration [2]. In a recent study, it was discovered that in Croatia and Hungary, fewer than 12% of children reported having at least one parent who had recently migrated for employment abroad, whereas in Romania and Albania, these percentages were 28% and 23%, respectively [5].

Parental migration is suggested to influence physical health, overall well-being and educational prospects of LBC. Studies have consistently shown that LBC, and particularly girls, exhibit lower subjective well-being compared to their non-LBC counterparts, and this disparity expands as they grow older [5–7]. Multiple systematic reviews have revealed an increased prevalence of behavioural problems, mental health disorders, and decreased well-being and coping abilities among LBC than among non-LBC [6, 8]. In contrast, some studies have contradicted these findings, indicating no differences in behavioural problems or mental health disorders [9, 10] and, in some cases, even suggesting better well-being and stronger personal psychological resources among LBC [9, 11]. However, most of this evidence originates from China, where parental migration predominantly involves rural-to-urban transitions [6, 8].

Furthermore, the existing body of evidence primarily relies on self-reported data from children, lacking input from the perspective of parents and caregivers. Notably, the health and well-being of LBC have gained attention in the scientific literature from low- and middle-income countries, as well as the Western Pacific region [12, 13]. Nevertheless, the mental health challenges of LBC are still not sufficiently studied and addressed in the European region. Despite studies conducted in Moldova [2, 7], Georgia [9, 14, 15] and Romania [16, 17] indicating a growing focus on the well-being of LBC [5], there remains a notable data gap from the Baltic countries, which limits our ability to effectively summarise and synthesise results for the region.

After regaining independence from the Soviet Union in 1990, Lithuania, a nation with a population of under 3 million, confronted persistent emigration challenges. A significant migration crisis emerged in 2010, with 80 thousand people leaving the country. Currently, annual migration stands at approximately 25 thousand individuals, with a notable number choosing to leave their children behind [18]. Labour migration has also become a source of income. According to World Bank estimates, remittances received from labour migration constituted over 4.5% in 2010, and currently account for more than 1% [19].

Concurrently, concerns persist regarding the mental well-being of Lithuanian children. Available data from Lithuania indicate a 12.1% prevalence of psychiatric disorders among adolescents aged 11–16, with conduct disorders (6.6%) and anxiety disorders (5.0%) being the most common categories [20]. Self-reported data from LBC show that those with migrant parents often experience heightened feelings of loneliness and longing for their absent parent. Additionally, these individuals exhibit an increased propensity for suicidal thoughts and self-harming behaviour [21]. Nevertheless, the existing evidence from Lithuania is limited in scope, lacking diverse perspectives and the latest findings.

In this study, we address the research gap in Baltic countries by evaluating the self-reported emotional and behavioural problems of LBC in Lithuania, while also comparing the reports provided by their parents or caregivers.

## Materials and methods

This cross-sectional study obtained ethical approval from the Vilnius Regional Biomedical Research Ethics Committee (No. 2021/11-1378-861). Prior to data collection, the Lithuanian Ministries of Education, Science and Sport, Health, Social Security and Labour, as well as municipalities, were informed about the research.

### Sampling

A total of 43 schools were invited to participate in the study, of which 24 agreed to take part. The final school list consisted of schools from 14 Lithuanian districts out of a total of 60 (Šiauliai City, Visaginas District, Pagėgiai District, Klaipėda City, Elektrėnai District, Klaipėda District, Vilnius City, Vilnius District, Trakai District, Panevėžys District, Panevėžys City, Radviliškis City, Marijampolės District, and Kretingos District). The school lists from each region were obtained from the Lithuanian Open Guidance System for Information and Consultation (AIKOS). The selection of invited schools was carried out using random numbers. Additionally, due to a higher-than-expected rejection rate, we also used direct contact with colleagues to enrol schools in the study.

All parents and children between the ages of 12 and 17 from the participating schools were invited to take part in this study. A priori sample size calculation using OpenEpi version 3.01 showed that the estimated sample size needed for a significance criterion α = 0.05 and expected power of 0.8 was 950 parents and 950 children.

### Data collection

Each selected school assigned a teacher, school psychologist, or social worker to be responsible for data collection. The first author (J.R.) conducted virtual training sessions and provided detailed instructions to the data collectors.

Informed consent was obtained from children and their parents or legal caregivers. Parents/caregivers completed the paper questionnaire at home and returned it to the data collector in a sealed envelope. Children whose parents/caregivers consented to their participation filled out the paper questionnaire in the classroom. To ensure anonymity, each child folded the completed questionnaire and sealed it.

The data collection took place between January 2022 and April 2023. Data entry was performed using EpiData (version 4.6.0.6).

### Instruments

The study used anonymous self-reported measures for parents/caregivers and their children. The authors developed a sociodemographic questionnaire that included questions about family status, self-reported health, and migration-related questions (Appendixes A, B).

The Achenbach System of Empirically Based Assessment (ASEBA) tools Child Behavioural Checklist (CBCL 6/18) and Youth Self-Report Questionnaire (YSR 11/18) were used to assess children’s emotional and behavioural problems. These ASEBA tools are widely used in cross-sectional studies due to their comprehensive and standardised approach. They also enable the possibility to collect information from multiple informants, including parents/caregivers, and directly from the children [22].

Participants were presented with three response options to reflect the child’s current state or experiences within the preceding six months (0 - not true, 1 - sometimes true, 2 - very true or often true). Scoring of these scales was carried out following the instructions outlined in the manual [22]. Notably, problem scales were excluded from scoring if they had more than eight missing items out of 113, excluding open-ended items and socially desirable items in YSR 11/18. There were 39 such cases in CBCL 6/18 and 35 in YSR 11/18 questionnaires.

The authors obtained a licence (No. 2334-07-23-21) for using CBCL 6/18 and YSR 11/18, previously translated and standardised for application within the Lithuanian population [23]. The Cronbach’s alpha values for the CBCL 6/18 and YSR 11/18 problem scales were > 0.72, and for the broadband scales (externalising and internalising), they were 0.9. The total score had a Cronbach’s alpha of 0.95. The validity, reliability and internal consistency of the CBCL 6/18 and YSR 11/18 questionnaires are fully described in a standardisation study [23].

### Statistical analysis

For statistical analysis, the children were categorised into two groups: left behind children with at least one migrant parent (LBC) and non-left behind children (non-LBC).

Complete case analysis was performed. We used descriptive statistics to determine frequencies in categorical data and calculate means along with 95% confidence intervals for continuous data. Comparative assessments were conducted using t tests to assess differences between LBC and non-LBC, as well as disparities between self-reported data from children and reports provided by parents/caregivers.

Associations between total scores and various sociodemographic factors were examined through bivariable linear regression. These factors included gender, child age, living place, LBC status, child sports performance, hobbies, and belonging to organisations as well as household chores and duties, number of close friends, child’s weekly frequency of interaction with friends outside of school, relationships with siblings, peers, and parents, individual problem-solving abilities, and school-related problems. Reduced regression models were constructed by excluding multicollinear variables (VIF > 5) and using a stepwise regression approach.

We conducted the statistical analysis using Stata software (version 15.1), and statistical significance was established with a *p* value < 0.05.

## Results

### Characteristics of participants

A total of 1400 children and 1400 parents/caregivers were invited to participate in the study. Among them, 735 children (a response rate of 52.5%) and 764 parents/caregivers (a response rate of 54.6%) agreed to participate and completed the questionnaire. However, four parents/caregivers and seven children corrupted the questionnaires, which were subsequently excluded. The final sample comprised 728 children and 760 parents or caregivers.

Among all children, 114 (15.7%) reported being LBC, 83.3% (*n* = 95) were left behind by their father, 9.7% (*n* = 11) were left behind by their mother, and 7% (*n* = 8) were left behind by both parents. In the parent/caregiver sample, 10.9% (*n* = 83) reported that children were LBC, 75.9% (*n* = 63) were left behind by their father, 9.6% (*n* = 8) were left behind by both parents, 4.8% (*n* = 8) were left behind by their mother and 9.6% (*n* = 8) had missing data. Table 1 presents detailed characteristics of the sample.

**Table 1:** Characteristics of participants: children (*n* = 728) and parents/caregivers (*n* = 760)

Characteristic		CHILDREN					PARENTS/CAREGIVERS				
		Total (*n* = 728)	LBC (*n* = 114)		Non-LBC (*n* = 587)		Total (*n* = 760)	LBC (*n* = 83)		Non-LBC (*n* = 509)	
		n	n	(%)	n	(%)	n	n	%	n	%
Child gender	Male	316	43	37.7	262	44.6	334	30	36.1	224	44.0
	Female	398	68	59.7	317	54.0	415	50	60.2	280	55.0
	*Missing*	14	3	2.6	8	1.4	11	3	3.6	5	1
Child age	12–13	217	26	22.8	182	31.0	193	17	20.5	138	27.1
	14–15	240	42	36.8	190	32.4	180	20	24.1	123	24.2
	16–17	216	32	28.1	177	30.2	167	18	21.7	120	23.6
	*Missing*	55	14	12.3	38	6.5	220	28	33.7	128	25.2
Living place	Rural	260	33	29	216	36.8	291	32	38.6	193	37.9
	Urban	459	80	70.2	367	62.5	457	49	59.0	315	61.9
	*Missing*	9	1	0.9	4	0.7	12	2	2.4	1	0.2
Number of child friends	0	24	3	2.6	20	3.4	20	0	0	14	2.8
	1	54	12	10.5	41	7	63	6	7.2	44	8.6
	2–3	302	46	40.4	247	42.1	357	38	45.8	243	47.7
	≥ 4	335	53	46.5	268	45.7	295	4	42.2	192	37.7
	*Missing*	13	0	0	11	1.9	25	4	4.8	16	3.1
The child’s weekly frequency of interaction with friends outside of school.	> 1	112	20	17.5	89	15.2	184	15	18.1	137	26.9
	1–2	220	32	28.1	181	30.8	259	37	44.6	165	32.4
	≥ 3	343	55	48.3	274	46.7	269	27	32.5	177	34.8
	*Missing*	53	7	6.1	43	7.3	48	4	4.8	30	5.9
Child relationship with siblings (compared to peers)	Worse	38	8	7.0	29	5	19	2	2.4	13	2.6
	Likewise	342	45	39.5	284	48.4	401	38	45.8	278	54.6
	Better	214	37	32.5	170	29	212	30	36.1	136	26.7
	No siblings	103	17	14.9	82	14	4	1	1.2	2	0.4
	*Missing*	31	7	6.1	22	3.8	124	12	14.5	80	15.7
Child relationship with peers (compared to peers)	Worse	36	6	5.3	29	5	19	2	2.4	14	2.8
	Likewise	451	74	64.9	359	61.2	512	50	60.2	352	69.2
	Better	206	28	24.6	174	29.6	176	27	32.5	115	22.6
	*Missing*	35	6	5.3	25	43	53	4	4.8	28	5.5
Child relationship with parents (compared to peers)	Worse	37	8	7.0	28	4.8	18	2	2.4	12	2.4
	Likewise	325	44	38.6	269	45.8	380	38	45.8	269	51.5
	Better	327	57	50	260	44.3	307	39	47.0	204	40.1
	*Missing*	39	5	4.4	30	5.1	55	4	4.8	31	6.1
Child do things by himself (compared to peers)	Worse	32	4	3.5	27	4.6	14	2	2.4	9	1.8
	Likewise	364	59	51.8	297	50.6	478	53	63.9	330	64.8
	Better	237	38	33.3	193	32.9	178	20	24.1	117	23
	*Missing*	95	13	11.4	70	11.9	90	8	9.6	53	10.4
Child performs in any sport	Yes	592	91	79.8	481	81.9	593	62	74.7	401	78.8
	No	126	22	19.3	99	16.9	153	17	20.5	102	20.0
	*Missing*	10	1	0.9	7	1.2	14	4	4.8	6	1.2
Child has hobbies	Yes	616	99	86.8	499	85.0	641	70	84.3	438	86.1
	No	97	13	11.4	78	13.3	94	9	10.8	60	11.8
	*Missing*	15	2	1.8	10	1.7	25	4	4.8	11	2.2
Child belong to any type of organization	Yes	230	30	26.3	193	32.9	265	22	26.5	197	38.7
	No	457	80	70.2	362	61.7	451	55	66.3	290	57
	*Missing*	41	4	3.5	32	5.5	44	6	7.2	22	4.3
Child has chores	Yes	369	51	44.7	310	52.8	406	49	59.0	286	56.2
	No	313	58	50.9	241	41.1	317	29	34.9	204	40.1
	*Missing*	46	5	4.4	36	6.1	37	5	6.02	19	3.7
Child has school related problems	Yes	284	50	43.9	227	38.7	55	6	7.23	40	7.9
	No	218	34	29.8	176	30.0	674	73	88.0	453	89.0
	*Missing*	226	30	26.3	184	31.4	31	4	4.8	16	3.1
Child has any type of illness of disability	Yes	44	12	10.5	29	4.9	43	6	7.2	27	5.3
	No	660	97	85.1	540	92	683	72	86.8	462	90.8
	*Missing*	24	5	4.4	18	3.1	34	5	6.0	20	3.9
Child health (compared to peers)	Perfect	230	32	28.1	188	32.0	275	30	36.1	183	36
	Good	360	50	43.9	299	50.9	415	42	50.6	289	56.8
	Satisfactory	114	27	23.7	84	14.3	56	9	10.8	33	6.5
	Bad	14	2	1.8	12	2.0	4	1	1.2	3	0.6
	Very bad	5	1	0.88	4	0.7	1	1	1.2	0	0
	*Missing*	5	2	1.8	0	0	9	0	0	1	0.2
How often child miss school due to illness	Once in a week	45	11	9.7	33	5.6	19	3	3.6	12	2.4
	Once in a month	222	39	34.2	177	30.2	177	29	34.9	117	23.0
	Once in a half year	318	43	37.7	263	44.8	383	33	39.8	262	51.5
	Once in a year	87	9	7.9	75	12.8	114	11	13.3	79	15.5
	Less than once in a year	51	11	9.7	38	6.5	54	6	7.2	34	6.7
	*Missing*	5	1	0.9	1	0.2	13	1	1.2	5	1.0

### Comparison of CBCL 6/18 and YSR 11/18 among LBC and non-LBC

Table 2 presents the results of the comparison of the YSR 11/18 and CBCL 6/18 problem scales, broadband scales, and total scores between LBC and non-LBC as well as the comparison of LBC self-reports and parent/caregiver reports. The mean comparison results revealed that, on average, LBC had significantly higher scores than non-LBC on all YSR 11/18 scales. In contrast, CBCL 6/18 scores showed no significant differences in means between LBC and non-LBC across any scale. However, LBC self-reported significantly higher scores across all scales than the reports provided by parents/caregivers.

**Table 2:** Emotional behavioural problems scales mean scores comparison between LBC and non-LBC and between child self-report and parent/caregiver report

Empirically based syndromes scales	YSR 11/18		CBCL 6/18		*p*- value*
	LBC (*n* = 110)	Non-LBC (*n* = 559)	LBC (*n* = 74)	Non-LBC (*N* = 492)	
	mean (95% CI)	mean (95% CI)	mean (95% CI)	mean (95% CI)	
Anxious/ Depressed	7.5 (6.4-8.5)	6.0 (5.6-6.5)	3.5 (2.6-4.3)	3.4 (3.1-3.8)	< 0.01
Withdrawn/ Depressed	4.6 (4.0-5.2)	3.7 (3.4-3.9)	2.1 (1.6-2.7)	2.3 (2.0-2.5)	< 0.01
Somatic Complains	5.1 (4.3-5.8)	3.8 (3.5-4.1)	3.0 (2.3-3.8)	2.8 (2.5-3.0)	0.02
Social Problems	5.4 (4.7-6.1)	4.1 (3.8-4.4)	2.2 (1.5-2.8)	2.1 (1.9-2.3)	< 0.01
Thought Problems	5.5 (4.6-6.4)	4.1 (3.8-4.5)	1.9 (1.1-2.7)	1.6 (1.4-1.8)	< 0.01
Attention Problems	6.3 (5.7-6.9)	5.4 (5.1-5.7)	3.5 (2.7-4.4)	3.6 (3.3-3.9)	< 0.01
Rule-Breaking Behaviour	4.8 (4.1-5.6)	3.8 (3.5-4.1)	2.1 (1.3-2.9)	1.9 (1.7-2.1)	< 0.01
Aggressive behaviour	5.9 (5.5-6.3)	7.0 (6.1-7.9)	3.8 (2.8-4.8)	4.0 (3.6-4.4)	< 0.01
Internalizing problems	17.1 (14.9-19.3)	13.5 (12.6-14.4)	8.7 (6.7-10.6)	8.5 (7.7-9.2)	< 0.01
Externalizing problems	11.9 (10.3-13.4)	9.7 (9.1-10.3)	5.9 (4.2-7.6)	5.9 (5.4-6.5)	< 0.01
Total score	57.7 (52.0-63.4)	47.1 (44.7-49.4)	24.9 (18.9-30.9)	24.1 (22.2-25.9)	< 0.01

* LBC YSR 11/18 and CBLC 6/18 comparison (t test)

### Associations between CBCL 6/18 and YSR 11/18 total scores and sociodemographic factors

We examined the associations between total YSR 11/18 and CBCL 6/18 emotional/behavioural problem scores in the bivariable linear regression analysis. The results indicated that being LBC (10.6, *p* < 0.01), being in the older age group (16–17 years old), and having a worse relationship with siblings were associated with higher YSR 11/18 total problem scores. Conversely, a higher frequency of weekly interaction with friends outside of school was associated with lower YSR 11/18 total problem scores. Notably, these variables did not significantly correlate with CBCL 6/18 total scores. Yet, unlike the YSR 11/18 problem scores, living in a rural area was associated with higher CBCL 6/18 total scores.

In both scales, higher total problem scores were associated with being female and having school-related problems. Conversely, having more close friends, having likewise relationships and better relationships with peers and parents, and doing things by oneself (independently) compared with peers were all associated with lower total problem scores. Table 3 presents detailed results of the bivariable linear regression.

**Table 3:** Bivariable linear regression for total emotional/behavioural problem score outcomes (YSR 11/18 and CBCL 6/18)

	YSR 11/18 total score		CBCL 6/18 total score	
Variables	Coefficient/constant*	*p* value	Coefficient/constant*	*p* value
**Gender**				
Male	Ref 40.5		Ref 17.8	
Female	13.4	**< 0.01**	3.9	**0.02**
**Child age**				
12–13	Ref 44.8		Ref 23.7	
14–15	3.9	0.16	1.6	0.69
16–17	6.5	**0.02**	1.4	0.56
**Living place**				
Rural	-2.5	0.27	4.1	**0.01**
Urban	Ref 49.2		Ref 22.2	
**LBC**				
Yes	10.6	**< 0.01**	0.8	0.77
No	Ref 47.1		Ref 24.1	
**Child perform any sport**				
Yes	-0.9	0.74	-0.3	0.88
No	Ref 48.9		Ref 24.1	
**Child has hobbies**				
Yes	0.52	0.87	-3.4	0.17
No	Ref 47.9		Ref 26.7	
**Child belong to any type of organization**				
Yes	3.2	0.18	-1. 0	0.55
No	Ref 47.75		Ref 24.2	
**Child has chores/duties at home**				
Yes	-1.9	0.40	-1.9	0.25
No	Ref 49.8		Ref 25.1	
**Number of child close friends**				
0	Ref 54.8		Ref 38.1	
≤ 1	2.9	0.68	-10.1	0.08
2–3	-2.1	0.74	-14.3	**0.01**
≥ 4	-12.5	**0.04**	-16.5	**< 0.01**
**The child’s weekly frequency of interaction with friends outside of school**				
< 1	Ref 57.7		Ref 24.7	
1–2	-11.3	**0.01**	-3.6	0.07
≥ 3	-10.2	**0.01**	-1.0	0.61
**Child relationship with siblings (compared to peers)**				
Worse	24.8	**< 0.01**	19.9	0.09
Likewise	1.0	0.76	-6.9	0.51
Better	-1.8	0.62	-10.6	0.32
No siblings	Ref 47.1		Ref 31.0	
**Child relationship with peers (compared to peers)**				
Worse	Ref 75.7		Ref 48.1	
Likewise	-28.6	**< 0.01**	-23.9	**< 0.01**
Better	-27.9	**< 0.01**	-27.6	**< 0.01**
**Child relationship with parents (compared to peers)**				
Worse	Ref 82.6		Ref 52.5	
Likewise	-36.5	**< 0.01**	-27.9	**< 0.01**
Better	-34.9	**< 0.01**	-31.5	**< 0.01**
**Child do things by himself (compared to peers)**				
Worse	Ref 62.6		Ref 47.4	
Likewise	-19.0	**< 0.01**	-23.4	**< 0.01**
Better	-5.7	0.30	-24.4	**< 0.01**
**Child has school related problems**				
Yes	23.7	**< 0.01**	17.6	**< 0.01**
No	Ref 37.4		Ref 22.4	

*Ref *p* < 0,01

Table 4 illustrates the results from multivariable linear regression reduced models for YSR 11/18 and CBCL 6/18 total emotional/behavioural problem scores. The complete multivariable regression model is presented in Appendix C.

In Model No. 1, we excluded multicollinear variables to prevent collinearity issues, and detailed results are presented in the table. Model No. 2 reflects the results after conducting stepwise selection.

For YSR 11/18 total problem scores, we observed significant associations with female gender, being LBC, and having school-related problems. In the CBCL 6/18 total problem scores, the following child factors remained significant: female gender, rural living place, and having school-related problems. Conversely, having hobbies was associated with lower CBCL 6/18 problem scores.

**Table 4:** Multivariable linear regression reduced models for YSR 11/18 and CBCL 6/18 total emotional/behavioural problem scores

	YSR 11/18				CBCL 6/18			
Variables	Model 1*		Model 2**		Model 1*		Model 2**	
	Coefficient (95% CI)	*p* value	Coefficient (95% CI)	*p* value	Coefficient (95% CI)	*p* value	Coefficient (95% CI)	*p* value
**Child gender**		**0.02**		**< 0.01**		**< 0.01**		**< 0.01**
Male	Ref		Ref		Ref		Ref	
Female	6.5 (0.9–12.1)		8.2 (3.2–13.1)		6.1 (1.8–10.4)		6.3 (2.2–10.5)	
**Child age group**		0.53				0.25		
12–13	Ref				Ref			
14–15	-1.9 (-8.2-4.4)				1.4 (-3.6-6.4)			
16–17	1.8 (-4.8-8.3)				-2.9 (-8.0-2.2)			
**Child living place**		0.76				**< 0.01**		**0.02**
Rural	-0.9 (-6.5-4.7)				5.7 (1.4–10.1)		5.2 (0.9–9.4)	
Urban	Ref				Ref		Ref	
**LBC**		0.11		**< 0.01**		0.66		
Yes	5.8 (-1.3-12.9)		8.9 (2.6–15.2)		1.4 (-4.9-7.7)			
No	Ref		Ref		Ref			
**Child perform any sport**		0.79				0.21		
Yes	1.1 (-6.7-8.8)				-3.5 (-9.1-2.0)			
No	Ref				Ref			
**Child has hobbies**		0.22				**0.02**		**0.02**
Yes	-5.4 (-14.1-3.2)				-8.8 (-16.0- -1.6)		-8.2 (-15.1- -1.2)	
No	Ref				Ref		Ref	
**Child belong to any type of organization**		0.05				0.89		
Yes	5.4 (0.1–10.9)				0.6 (-3.7-4.9)			
No	Ref				Ref			
**Child has chores/duties at home**		0.34				0.66		
Yes	-2.7 (-8.1-2.7)				0.9 (-3.3-5.1)			
No	Ref				Ref			
**The child’s weekly frequency of interaction with friends outside of school**		0.06				0.59		
< 1	Ref				Ref			
1–2	-9.1 (-16.6- -1.5)				-1.7 (-6.7-3.4)			
≥ 3	-5.6 (-12.6-1.7)				0.8 (-4.3-5.9)			
**Child relationship with siblings (compared to peers)**		0.12						
No siblings	Ref							
Worse	12.7 (-0.5-25.9)							
Likewise	2.7 (-5.1-10.5)							
Better	-0.4 (-8.7-7.9)							
**Child has school related problems**		**< 0.01**		**< 0.01**		**< 0.01**		**< 0.01**
Yes	20.7 (15.3-26.1)		21.5 (16.6-26.4)		18.9 (11.9-25.8)		19.3 (12.4-26.2)	
No	Ref		Ref		Ref		Ref	
**Constant**	36.1 (20.-–51.2)	< 0.01	24.2 (16.0-32.4)	< 0.01	21.5 (10.1-32.9)	< 0.01	17.9 (8.6-27.3)	< 0.01
**R** ^**2**^	0.22		0.20		0.12		0.10	
**Adj. R** ^**2**^	0.20		0.20		0.09		0.9	
**F**	7.12		38.7		4		10.7	
***p*** ** value of the model**	< 0.01		< 0.01		< 0.01		< 0.01	

* after removing variables due to multicollinearity (VIF > 5)

** after conducting stepwise selection

## Discussion

This study assessed the emotional and behavioural problems experienced by LBC and non-LBC in Lithuania, also comparing their self-reported experiences with reports provided by parents or caregivers. The findings underscore that, in contrast to their non-LBC peers, LBC consistently report more frequent anxiety, withdrawal, depression, somatic problems, difficulties in social interaction, thought patterns, attention, rule-breaking, and aggressive behaviour. Specifically, being a female, being LBC, and encountering school-related problems were identified as factors associated with heightened emotional and behavioural difficulties in our study sample. When comparing the self-reports of children with the reports from their parents or caregivers, it became evident that LBC often reported more emotional and behavioural challenges than parents or caregivers perceived. However, there were no notable differences in the reports between LBC and non-LBC regarding assessments provided by parents or caregivers.

The findings of our study align with prior research in this domain, indicating that LBC are at heightened risk of experiencing emotional and behavioural problems, along with diminished well-being when compared to their non-LBC counterparts [5, 6, 8, 24]. Our study, echoing previous research in Lithuania, draws attention to the fact that LBC frequently report a higher prevalence of adverse effects on their mental health and well-being. This, in turn, may contribute to behavioural and emotional challenges [21]. In contrast, in Georgia, several earlier studies have indicated that the migration of a family member did not result in substantially higher total difficulty scores in LBC [15, 25], highlighting disparities within and across different regions [8, 9].

Our study additionally incorporates perspectives from multiple informants. Notably, our findings reveal a trend wherein LBC consistently report higher scores compared to their parents or caregivers. While LBC self-reports indicate higher emotional and behavioural problems compared to non-LBC peers, these distinctions do not emerge in the reports provided by parents or caregivers. This phenomenon is widespread across various societies, though the extent of the informant effect exhibits variability contingent upon factors such as ethnicity, religion, cultural values, historical background, geographical location, educational levels, political climate, and economic conditions [26]. Our results underscore children as being valuable sources of information, especially during adolescence, as corroborated by previous research [27]. Moreover, prior studies confirm that emotional issues reported by adolescents themselves are more dependable than reports from their caregivers [27–29]. Despite prior research indicating a medium correlation coefficient of approximately 0.25 between parental and children’s responses in ASEBA questionnaires [30] the observed differences may also be attributed to specific characteristics unique to the participants of this study. First of all, the destigmatisation of mental health issues has recently begun in Lithuania; however, adults may still be reluctant to acknowledge and report emotional and behavioural problems in their children. Secondly, the majority of parental/caregiver reports were predominantly provided by one caregiver, with the other being absent due to emigration. The parent/caregiver might felt a sense of guilt for problems arising from parental emigration, leading to underreporting the child’s mental health concerns. In the existing literature on LBC, reliance is often placed on reports from teachers, parents or caregivers, or the children themselves. As noted by Achenbach et al., divergences among informants’ underscore disparities in assessments of child functioning across distinct contextual situations, so it is crucial collect information from as many informants as possible [31, 32]. To the best of our knowledge, this study is one of the first attempts on this topic to collect information from multiple informants.

Additionally, our study highlights the influence of sociodemographic characteristics on LBC child well-being. For example, we find that among LBC, girls exhibit greater vulnerability and report more emotional problems than LBC boys. This gender-based disparity is evident in both the children’s self-reports and the reports provided by parents or caregivers. Gender-related differences have also been observed in previous studies from Eastern European countries [5, 7, 16]. Notably, this gender disparity extends beyond the LBC population, prevailing throughout the broader adolescent population in Eastern Europe, with girls exhibiting greater vulnerability than boys [25]. However, greater gender disparity in LBC well-being may be rooted in the unique circumstances of migration contexts. Girls separated from their migrant parents often shoulder increased responsibilities at home, including caring for younger siblings or other household tasks [33]. Family-related factors, including single parenthood, unfavourable family climate, and challenges in disciplinary practices, were found to be associated with an elevated incidence of mental health problems among Lithuanian children [20]. A prior study conducted in Lithuania identified a distinct profile among children with parents in emigration, despite encountering challenges similar to those in divorced families [21]. Upon conducting a primary analysis of our study data, we observed that the parental family status did not apply a significant influence on the final results. Consequently, we made the decision to exclude family status from further analysis. This decision aligns with findings from a meta-analysis, which indicated that family relationship was a more important factor than family type. In mentioned survey joint custody, wherein both parents remain actively involved in the child’s life despite not cohabiting, serves as a protective factor, contributing to child resilience [34].

In this survey, the LBC group predominantly consisted of children left behind by their fathers, with the number of respondents in other subgroups comparatively low. Results from previous studies on the gender of the migrant parent present contradictory findings. On one hand, there is evidence suggesting that a greater negative impact is expected when the father is absent [10, 35]. On the other hand, other studies argue that children with a mother in emigration are more vulnerable [36] while some research found no difference in impact when one or both parents are in migration [37, 38]. Finally, meta-analytic evidence indicates that parent migration itself, regardless of whether one or both parents migrate, has a significant impact on the physical and mental health of children [6].

The potential impact of the COVID-19 pandemic on our results cannot be overlooked. Rajmil L. and colleagues synthesised evidence from 22 studies encompassing different income-level countries, revealing a decline in the mental well-being of children and adolescents across diverse geographical and socioeconomic contexts, alongside reduced physical activity and increased sedentary behaviours as an impact of lockdown [39]. A study conducted in Lithuania assessing the effects of pandemic-related quarantine, school closures, and remote learning on younger school-age children highlighted an increased prevalence of somatic complaints due to extended screen time [40]. Additionally, various studies have documented heightened mental health challenges among adolescents during the pandemic [41]. Meanwhile, qualitative research has indicated that children perceive the COVID-19 pandemic as challenging. LBC display improved coping abilities with lockdown measures, particularly those with close relationships with returned parents and siblings and a higher socioeconomic status [42]. Notably, all children in our study were equally exposed to lockdown measures and remote learning. Despite this shared exposure, LBC reported more emotional and behavioural symptoms. This discrepancy may account for our study sample’s relatively elevated mean total scores compared to a representative Lithuanian sample [30]. These findings emphasise the unique challenges faced by LBC during the pandemic.

The strength of this study lies in the comprehensive approach to analysing data from multiple informants, including children and parents/caregivers, enabling a multifaceted interpretation of the results. Adhering to the COVID-19 pandemic regulations in Lithuania, the initial round of data collection restricted external access to schools; thus, questionnaires were administered by familiar and trusted school personnel trained for this purpose. Implementing stringent confidentiality measures through anonymous questionnaires, sealed envelopes, and adhesives further fostered a willingness among participants to complete the questionnaires thoroughly.

This study has several limitations. Despite data collection spanning various regions in Lithuania, the absence of a representative sample restricts the generalisability of our findings to the entire country. Only a subset of the schools was randomly selected and invited to participate in the study. Robustness checks mitigated potential sampling impact on the overall results concerning how the schools were chosen for the study. Additionally, we were not able to reach the estimated sample size. The relatively low response rate from children and their caregivers could be attributed to data collection challenges stemming from the COVID-19 pandemic, where fluctuating school attendance and temporary closures were prevalent due to heightened infectious disease concerns. This pandemic-related context could have also influenced children’s emotional well-being and consequently impacted the overall study results. Furthermore, the possibility of bias exists due to the exclusion of individuals with missing values and the option for respondents to decline participation. This may introduce a bias towards including only highly motivated participants in the study. Moreover, the questionnaires address highly sensitive issues concerning parents’ emigration and children’s emotional and behavioural challenges. It is highly plausible that the reluctance of children with severe issues and their parents / caregivers to participate may be attributed to the sensitive nature of these inquiries. Finally, the study’s cross-sectional design prevents us from drawing conclusions about the potential origins of the emotional and behavioural difficulties of LBC.

The findings of our study highlight the need for more comprehensive nationally representative research in Eastern European countries to fully comprehend the impact of parental emigration on the mental well-being of their LBC, thereby delineating the prevailing situation in the region. The disparities observed between parental or caregiver perspectives and children’s self-reports emphasise the imperative for incorporating multiple sources of information in forthcoming studies. It is essential to direct parental attention toward the emotional health of their children through evidence-based parenting programmes while simultaneously enhancing parental mental health literacy to facilitate an understanding and recognition of their children’s challenges. Furthermore, creating a supportive environment for LBC not only involves parents but also extends to caregivers, teachers, school psychologists, and healthcare providers who can be equipped with the necessary skills to offer emotional and psychological aid. In the end, the issues concerning left behind children can only be fully fixed by either helping parents work where they currently live or assisting children in moving with their parents [12]. However, these solutions demand extensive collaboration across various sectors and nations. In the interim, ensuring the well-being of LBC mandates significant input from parents, guardians, communities, educators, policymakers, and service providers [3].

## Conclusion

Our study observed that LBC self-reported higher emotional and behavioural difficulties than their non-LBC peers. Results also reveal a disparity between the reports provided by parents or caregivers and those of children, with parents/caregivers indicating lower problem scores. Findings of this study, also uncovered key factors impacting the emotional and behavioural outcomes of LBC, including living environment, school-related concerns, and hobbies. These factors highlight the multifaced nature of LBC lived experiences. Overall, our study highlights the need for further research in Eastern European countries and emphasises the importance of a broad supportive network to ensure the emotional well-being of LBC. Such networks should encompass parents, caregivers, school personnel, and the community to address the multifaceted challenges that LBC may encounter and ensure their emotional health and well-being.

### Electronic supplementary material

Below is the link to the electronic supplementary material.

Supplementary Material 1

Supplementary Material 2

Supplementary Material 3

## Data Availability

Data are stored in the Vilnius University Faculty of Medicine Institute of Health Sciences Department of Public Health in compliance with relevant data protection laws. Datasets of the current study are available upon request to the corresponding author.

## Abbreviations

LBC: Children left behind in the home country due to at least one parent emigration
AIKOS: Lithuanian open guidance system for information and consultation
ASEBA: The Achenbach System of Empirically Based Assessment
CBCL 6/18: Child Behavioural Checklist (Achenbach, 1991)
YSR 11/18: Youth Self-Report (Achenbach, 1991)
UNICEF: United Nations Children’s Fund
