# Supplementary material for: Emotional and behavioural problems of left behind children in Lithuania: a comparative analysis of youth self-reports and parent/caregiver reports using ASEBA
Source: Child Adolesc Psychiatry Ment Health. 2024 Mar 18;18:33. doi: 10.1186/s13034-024-00726-y (PMC10949819; doi:10.1186/s13034-024-00726-y)
Supplement: Supplementary file 1 — Supplementary Material 1 [file 13034_2024_726_MOESM1_ESM.docx]

**Appendix A. Additional questionnaire for parents**

**1. What is the height of the child who brought the questionnaire?**__ __ __ cm

**2. What is the weight of the child who brought the questionnaire?**__ __ kg

**3. Where do you live?**
□ In a country house
□ In a village
□ In a city
□ In a big city

**4. How many people live in your house?**□ Enter the number: ___

**5. How many of the people living in the house are children under the age of 18?**□ Enter the number: ___

**6. What is your marital status:**
□ Single
□ Married
□ Living together outside of marriage
□ Divorced
□ Widowed

**7. Compared to other children of this age, how would you describe the health of this child?**□ Excellent
□ Good
□ Satisfactory
□ Bad
□ Very bad

**8. How often does the child miss lessons due to illness?**
□ Once a week
□ Once a month
□ Once every six months
□ Once a year
□ Less than once a year

**9. Are you a caregiver of this child while his parents/one of the parents is abroad?**□ Yes
□ No (go to the questions on the next page)

**10. Which parent is away?**□ Both parents
□ Mother
□ Father

**11. How long have both parents or one of the parents lived abroad?**
**Mother**
□ Years: (enter number)_______
□ Months: (enter number)_______
**Father**
□ Years: (enter number)_______
□ Months: (enter number)_______

**12. How often do the child's parents return to Lithuania?**□ Once a month/several months
□ Once every six months
□ Once a year
□ Once every two years and less often
□ Never returned/does not return

**13. Does the child go to visit the parents/one of the parents abroad?**□ Yes
□ No (go to question 15)

**14. How often does the child go to visit the parents/one of the parents abroad?**□ Once a month/several months
□ Once every six months
□ Once a year
□ Other (enter)___________

**15. Does the child communicate with his parents remotely (Skype, Messenger, Viber, etc.)?**□ Yes
□ No (go to questions on next page)

**16. How often does the child communicate with parents abroad?**□ Daily
□ 1-2 times a week
□ 1-2 times a month
□ 1-2 times a half year
□ Other (enter)______________________________

**17. Do the child's parents send you remittances from abroad?**□ Yes
□ No
