# Supplementary material for: Emotional and behavioural problems of left behind children in Lithuania: a comparative analysis of youth self-reports and parent/caregiver reports using ASEBA
Source: Child Adolesc Psychiatry Ment Health. 2024 Mar 18;18:33. doi: 10.1186/s13034-024-00726-y (PMC10949819; doi:10.1186/s13034-024-00726-y)
Supplement: Supplementary file 2 — Supplementary Material 2 [file 13034_2024_726_MOESM2_ESM.docx]

**Appendix B. Additional questionnaire for children**

**1. How tall are you?**__ __ __ cm

**2. How much do you weigh?**__ __ kg

**3. Where do you live?**
□ In a country house
□ In a village
□ In a city
□ In a big city

**4. How many siblings do you have?**□ I don’t have siblings
□ I have brothers (enter number)____
□ I have sisters (insert number)____

**5. Compared to your classmates, how would you describe your health?**□ Excellent
□ Good
□ Satisfactory
□ Bad
□ Very bad

**6. How often do you miss classes due to illness?**
□ Once a week
□ Once a month
□ Once every six months
□ Once a year
□ Less than once a year

**7. You have:**
□ Both parents
□ Only father
□ Only mother
□ I have neither father nor mother (go to the questions on the next page)

**8. Who do you live with?**
□ I live with both parents
□ I live only with my mother
□ I live only with my father
□ I live only with my grandparents
□ I live only with relatives
□ I live with guardians
□ I live alone

**9. Have your parents or one of your parents gone abroad?**□ Yes
□ No (go to the questions on the next page) **10. Which parent is abroad?**□ Mother
□ Father
□ Both parents

**11. How long have both parents, father or mother, lived abroad?
Mom**
□ Years: (enter number)_______
□ Months: (enter number)_______  **Dad**
□ Years: (enter number)_______
□ Months: (enter number)_______

**12. How often do parents return to Lithuania?**□ Once a month/several months
□ Once every six months
□ Once a year
□ Once every two years and less often
□ Never returned/does not return

**13. Do you go to visit your parents/one of your parents?**□ Yes
□ No (Go to question 15)

**14. How often do you go to visit your parents/one of your parents?**□ Once a month/several months
□ Once every six months
□ Once a year
□ Other (write)_________________

**15. Do you communicate with your parents remotely (Skype, Messenger, Viber, etc.)?**□ Yes
□ No (go to the questions on the next page)

**16. How often do you communicate with your parents at a distance?**□ Daily
□ 1-2 times a week
□ 1-2 times a month
□ 1-2 times a half year
□ Other (write)______________________________
