# Supplementary material for: Emotional and behavioural problems of left behind children in Lithuania: a comparative analysis of youth self-reports and parent/caregiver reports using ASEBA
Source: Child Adolesc Psychiatry Ment Health. 2024 Mar 18;18:33. doi: 10.1186/s13034-024-00726-y (PMC10949819; doi:10.1186/s13034-024-00726-y)
Supplement: Supplementary file 3 — Supplementary Material 3 [file 13034_2024_726_MOESM3_ESM.docx]

**Appendix C.** Multivariable linear regression for YSR 11/18 total emotional and behavioural total problem scores with all variables from binary regression included.

| **Variables** | **YSR 11/18 total score** | | **CBCL 6/18 total score** | |
| --- | --- | --- | --- | --- |
|  | **Coefficient**  **(95% CI)** | **p value** | **Coefficient**  **(95% CI)** | **p value** |
| **Child gender** |  | 0.03 |  | <0.01 |
| Male | Ref |  | Ref |  |
| Female | 6.5 (1.0-12.1) |  | 8.05 (3.5-12.6) |  |
| **Child age group** | | 0.63 |  | 0.37 |
| 12-13 | Ref |  | Ref |  |
| 14-15 | -2.3 (-8.6-4.0) |  | 2.0 (-3.4-7.4) |  |
| 16-17 | 0.5 (-6.1-7.1) |  | -1.9 (-7.2-3.4) |  |
| **Child living place** |  | 0.81 |  | <0.01 |
| Rural | -0.7 (-6.1-4.8) |  | 7.1 (2.6-11.7) |  |
| Urban | Ref |  | Ref |  |
| **LBC** |  | 0.06 |  | 0.51 |
| Yes | 6.8 (-0.2-13.8) |  | 2.3 (-4.7-9.3) |  |
| No | Ref |  | Ref |  |
| **Child perform any sport** |  | 0.16 |  | 0.34 |
| Yes | 5.7 (-2.2-13.7) |  | -2.8 (-8.7-2.9) |  |
| No | Ref |  | Ref |  |
| **Child has hobbies** |  | 0.25 |  | <0.01 |
| Yes | -5.1 (-13.8-3.6) |  | -10.0 (-17.4- -2.5) |  |
| No | Ref |  | Ref |  |
| **Child belong to any type of organization** |  | 0.31 |  | 0.66 |
| Yes | 2.8 (-2.6-8.1) |  | 1.0 (-3.5-5.5) |  |
| No | Ref |  | Ref |  |
| **Child has chores/duties at home** |  | 0.61 |  | 0.46 |
| Yes | -1.4 (-6.8-4.0) |  | 1.7 (-2.9-6.3) |  |
| No | Ref |  | Ref |  |
| **Number of child**  **close friends** | | 0.19 |  | 0.84 |
| 0 | Ref |  | Ref |  |
| ≤1 | 11.5 (-16.2-39.2) |  | -4.3 (-31.6-23.0) |  |
| 2-3 | 15.6 (-10.5-41.6) |  | -5.0 (-31.8-21.7) |  |
| ≥4 | 10.4 (-16.1-36.9) |  | -6.9 (-33.8-20.1) |  |
| **The child's weekly frequency**  **of interaction with friends**  **outside of school** | | 0.18 |  | 0.15 |
| < 1 | Ref |  | Ref |  |
| 1-2 | -6.3 (-13.8-1.3) |  | -2.3 (-7.8-3.2) |  |
| ≥ 3 | -1.8 (-8.9-5.5) |  | 2.7 (-2.8-8.2) |  |
| **Child relationship with**  **siblings (compared to peers)** | | 0.83 |  | <0.01 |
| No siblings | Ref |  | <5 observations |  |
| Worse | 2.7 (-10.6-16.0) |  | Ref |  |
| Likewise | 3.1 (-5.2-11.3) |  | -21.1 (-33.2- -8.9) |  |
| Better | 0.6 (-8.3-9.5) |  | -19.5 (-32.4- -32.4- -6.7) |  |
| **Child relationship with**  **peers (compared to peers)** | | 0.04 |  | <0.01 |
| Worse | Ref |  | Ref |  |
| Likewise | -14.8 (-26.5- -3.1) |  | -26.0 (-38.7- -13.4) |  |
| Better | -14.8 (-27.2- -2.5) |  | -27.3 (-40.8- -13.8) |  |
| **Child relationship with**  **parents (compared to peers)** | | <0.01 |  | 0.04 |
| Worse | Ref |  | Ref |  |
| Likewise | -22.9 (-33.9- -11.9) |  | -13.3 (-27.4-0.7) |  |
| Better | -21.8 (-32.8- -10.9) |  | -17.3 (-31.8- -2.8) |  |
| **Child do things by**  **himself (compared to peers)** | | <0.01 |  | <0.01 |
| Worse | Ref |  | Ref |  |
| Likewise | -22.6 (-36.1- -9.1) |  | -40.1 (-56.8- -23.3) |  |
| Better | -15.1 (-28.8 - -1.5) |  | -38.4 (-55.7- -21.1) |  |
| **Child has concerns or problems related with school** |  | <0.01 |  | <0.01 |
| Yes | 1.7 (11.1-22.3) |  | 16.0 (8.7-23.4) |  |
| No | Ref |  | Ref |  |
| **Constant** | 73.2 (42.9-103.4) | <0.01 | 121.9 (86.4-157.6) | <0.01 |
| **R^2^** | 0.34 | | 0.32 | |
| **Adj. R^2^** | 0.29 | | 0.27 | |
| **F-statistic** | 6.95 | | 18.2 | |
| **p value of the model** | <0.01 | | <0.01 | |
